# The Influence of an Intense Training Regime in Professional and Non-Professional Athletes on Semen Parameters: A Systematic Review

**DOI:** 10.3390/jcm14010201

**Published:** 2025-01-01

**Authors:** Francesca Greco, Giovanni Guarascio, Elisa Giannetta, Francesco Pio Oranges, Federico Quinzi, Gian Pietro Emerenziani, Maria Grazia Tarsitano

**Affiliations:** 1 Department of Experimental and Clinical Medicine, University “Magna Græcia” of Catanzaro, 88100 Catanzaro, Italy; f.greco@unicz.it (F.G.); giovanni.guarascio002@studenti.unicz.it (G.G.); francescopio.oranges@studenti.unicz.it (F.P.O.); fquinzi@unicz.it (F.Q.); 2 Department of Experimental Medicine, Sapienza University of Rome, Viale Regina Elena 324, 00161 Rome, Italy; elisa.giannetta@uniroma1.it; 3 Department of Neuroscience, Biomedicine and Movement, University of Verona, 37124 Verona, Italy; 4 Department of Human Science and Promotion of Quality of Life, San Raffaele Open University of Rome, 00166 Rome, Italy; mariagrazia.tarsitano@uniroma5.it

**Keywords:** athletes elite, male fertility, semen quality, sperm parameters, trained individuals

## Abstract

**Background/Objectives**: Male infertility is influenced by physiological factors like age, as well as lifestyle factors, including physical activity. However, the specific impact of sport activity on semen parameters, and thus on male fertility, remains unclear. Specifically, the aim of this systematic review is to evaluate how an intense regime of training may affect sperm parameters in professional and non-profession athletes. **Methods:** Studies reporting sperm parameters associated with high training load were included. In April 2024, three electronic databases and literature sources (PubMed, Scopus, and Web of Science) were searched. Quality appraisal was performed independently by three authors using the National Heart, Lung, and Blood Institute Quality Assessment Tools (NHLBI-QAT). **Results**: Four studies met the inclusion criteria, reporting a total of 156 participants. Sixteen weeks of intensive cycling training produced a significant decrease in seminal volume, sperm concentration, sperm motility, and morphology, with a return to their initial levels, except for sperm morphology and sperm concentration, after at least one week of rest. In addition, in athletes with varicocele, a 6-month stop from sports activity went a long way toward improving sperm concentration and sperm motility. However, DNA fragmentation, a greater presence of round cells, and high numbers of active macrophages were described. At least 30 days improve semen parameters in professional and non-professional athletes. **Conclusions:** Intensive training could worsen seminal parameters and, consequently, male fertility. However, certainty of evidence is very low, and the results should be interpreted with caution.

## 1. Introduction

A significant increase in male infertility has been observed in recent decades [1], thus becoming a high priority for many national and international health organizations [1]. According to the World Health Organization (WHO) report, a large number of people are affected by infertility during their lifetime. About 17.5% of the adult population present with infertility, and the male partner is responsible for 50% of cases of infertility in couples [2,3]. Infertility rates are similar between high-income and low-income countries [2].

Thus, access to affordable, high-quality fertility care for those individuals is needed [2]. Male infertility or reduced fertility can result from testicular dysfunction [4], endocrinopathies [5], lifestyle factors [6], congenital anatomical factors [7], gonadotoxic exposures [8], and ageing [9], among others [10]. Spermatogenesis is controlled by testosterone and follicle-stimulating hormone (FSH) [11]. FSH is released by the pituitary gland and stimulates primary spermatocytes to undergo the first meiotic division [11]. Testosterone, produced and released by Leydig cells in the testes under the regulation of luteinizing hormone (LH), promotes the development of secondary spermatocytes into mature spermatozoa [11]. As the quality of semen plays an important role in male infertility, its analysis becomes crucial [12].

According to the 2021 guidelines from the WHO [13], the critical parameters for assessing semen quality have been defined to ensure accurate and consistent diagnosis of male infertility (see Table 1). These guidelines emphasize the importance of standardized procedures to ensure that results are consistent and reliable across different laboratories, thereby aiding in the accurate diagnosis and treatment of male infertility [13,14].

Various factors can cause abnormal seminal parameters, including varicocele, infections of the accessory glands, immunological issues, congenital defects, obstructive azoospermia, and conditions related to medical treatments, systemic diseases, and endocrine disorders [12,15].

According to the 2021 WHO guidelines [13], in addition to the traditional parameters measured in semen analysis (e.g., volume, motility, morphology, and concentration), several advanced and accessory measurements are considered important for a comprehensive assessment of male fertility. These additional parameters include sperm DNA fragmentation and reactive oxygen species (ROS) levels [13,14]. Sperm DNA fragmentation refers to alterations in the normal chemical structure of DNA, including breaks in DNA strands within the sperm nucleus [13]. Regarding sperm DNA fragmentation, multiple studies published in recent years indicate that sperm DNA fragmentation can affect embryonic development, implantation, and pregnancy in both natural and assisted reproduction [16,17]. It is also known that sperm DNA fragmentation is predominantly found in men with altered semen parameters [18].

Regarding the ROS levels, there is abundant evidence to support the important role of oxidative stress in the onset of male infertility [18,19,20,21]. ROS are generated primarily by leukocytes, abnormal and immature spermatozoa, and as a result of oxidative metabolic pathways and cytosolic and plasma membrane oxidases [22,23,24]. While controlled amounts of ROS are essential for normal cellular physiological functions, including spermatogenesis and spermatic functions involved in fertilization [23,25,26,27], an imbalance with excess ROS relative to antioxidants can lead to oxidative stress. The latter can cause damage to the seminal fluid membrane and DNA of spermatozoa, reducing their ability to fertilize an egg and generate a healthy embryo [23,28,29,30,31]. These results underscore the importance of maintaining a balance between ROS and antioxidants to preserve the male reproductive health. With advancing age, testosterone levels in men decrease and hypogonadism can occur. Seminal fluid parameters like volume and motility decline as early as age 35 [32], and morphology may also become increasingly abnormal [33]. After the age of 40, men may suffer damage to their sperm DNA, there may be a decline in motility (40%) and viability (below 50%) [9].

Regarding lifestyle factors, several studies indicate the beneficial effects that physical activity could have on different aspects of overall health status [34,35,36] and even to maintain adequate reproductive and sexual health [37]. Nevertheless, the positive effects of an active lifestyle on spermatogenesis were confirmed by comparing the sperm parameters of physically active and sedentary men [38]. Indeed, higher levels of FSH, LH and testosterone were reported in physically active individuals compared with sedentary individuals [38]. Type, intensity, volume, and frequency of physical activity could differently influence the semen parameters [39]. Regular, moderate exercise training appears to be beneficial for oxidative stress and health. In contrast, acute exercise, experienced by professional and non-professional athletes, may increase oxidative stress [40].

In the field of exercise endocrinology, it is crucial to differentiate between the immediate impact of a single exercise session on hormone levels (e.g., testosterone, growth hormone) and the longer-term effects of regular exercise training. During an acute exercise session, various forms of physical activity typically lead to alterations in circulating hormone concentrations (testosterone and growth hormone), almost universally resulting in increased levels [41,42,43,44,45]. These changes are usually proportional to the intensity and duration of the exercise, although the specific mode of exercise (like swimming, running, or weightlifting) may influence the degree of response [41,42]. However, these acute changes tend to subside relatively quickly during the recovery period, unless the exercise session is exceptionally prolonged (e.g., lasting for several hours) [43]. Conversely, prolonged or intense chronic exercise, like marathon or long-distance running, may lead to hypogonadism [44,45]. Indeed, prolonged or intense exercise, particularly in endurance athletes, may result in a notable decrease in testosterone levels over time, while short-term bouts of exercise typically cause a temporary rise in testosterone levels [46]. Excessive endurance training can disrupt the normal functioning of the hypothalamic–pituitary–gonadal axis, resulting in sustained reductions in basal resting testosterone levels to the lower end of the age-appropriate normal range [44]. This phenomenon, known as exercise-induced hypogonadal male condition, may have significant clinical implications due to the decreased testosterone levels [44].

Endurance exercise, including activities like long-distance running or extensive cycling, has been observed to potentially worsen seminal parameters [47]. Specifically, activities like cycling could lead deteriorations in seminal parameters [48,49,50]. The main aim of physical exercise is to enhance the physical performance of practitioners regardless of their performance level. However, when training becomes prolonged and excessive, or when recovery is insufficient or poorly managed, many of the physiological changes associated with physical training are reversed, leading to a state known as overreaching/overtraining [51]. Multiple studies have reported repeated negative consequences on spermatogenesis following intense training [49,52]. While physical activity may have positive effects on male fertility, the effect of high training load had to be clarified. The aim of this systematic review is to evaluate the impact of high training load (typical of elite athletes) on male fertility, with a specific focus on semen parameters.

## 2. Materials and Methods

### 2.1. Information Sources

This systematic review was conducted following the guidelines established by the Preferred Reporting Items for Systematic Reviews and the Meta-Analyses (PRISMA) statement [53]. It was registered in PROSPERO the International Prospective Register of Systematic Reviews, with code ID CRD42024539754.

### 2.2. Search Strategy

A systematic search of three electronic databases (PubMed, Scopus, Web of Science/Core Collection) was performed using predefined search terms deduced from eligibility criteria on the 10 April 2024. The reference lists of identified reviews and included articles were hand searched for potentially relevant articles. The search was conducted using specific keywords (Table 2).

### 2.3. Study Selection and Data Extraction

Following the initial data search, two authors of this study (G.G and F.P.O.) independently evaluated the titles and abstracts of all the articles previously identified using the search strategy and screened them for eligibility according to predefined inclusion and exclusion criteria. In the case of hesitancy about the inclusion of a study, the two independent reviewers discussed the merits of selection. If a consensus could not be reached, a third reviewer (MGT) was consulted to resolve the issue and an agreement was achieved. After this initial data search, the included studies were read in full and the following article data were extracted: title, author, year of publication, number and age of participants, type of sport, purpose, fertility outcomes, and summary of results.

### 2.4. Eligibility Criteria

Studies were further analyzed and considered eligible if the following inclusion criteria were met: male professional and non-professional athletes with at least three training sessions per week with high intensity training; written in the English language; published in the last 10 years; clinical trials and observational studies. Reports on animals and in vitro studies were excluded. Moreover, systematic reviews and duplicated publications were excluded. All the studies resulting from the search were reported on an electronic spreadsheet and duplicates were removed using the Rayyan [54]. Studies were further analyzed and deemed eligible according to the following inclusion criteria: (a) clinical trials and observational studies; (b) a focus on sperm parameters and sport activity; (c) male professional and non-professional athletes with at least three training/week with high intensity training; (d) men without chronic illnesses or fertility problems; (e) published in English; (f) published in the last 10 years. The exclusion criteria were as follows: (a) review articles; (b) book chapters; (c) studies using animal and in vitro models; (d) studies not addressing the effect of sport activity on sperm parameters; and (e) studies based and (f) duplicated.

### 2.5. Quality Assessment and Risk of Bias Tool

All studies were checked for methodological quality using a checklist to satisfy the present topic. Quality appraisal was performed independently by three authors using the National Heart, Lung, and Blood Institute Quality Assessment Tools (NHLBI-QAT) [55]. A total of 14 criteria were scored. For each criterion, 1 point was assigned to the study that met the criteria (“yes”) and, 0 points were assigned if the study did not meet the criteria (“no” or “cannot determine, not applicable, not reported”). Based on these scores, we obtained an overall rating, determining each study’s quality as poor, fair, or good. The questions on the assessment tool were designed to help reviewers focus on the key concepts for evaluating a study’s internal validity. They are not intended to create a list that is simply tallied up to arrive at a summary judgement of quality. The quality assessment results of each study are reported in (Supplementary Materials).

## 3. Results

**Figure 1:**
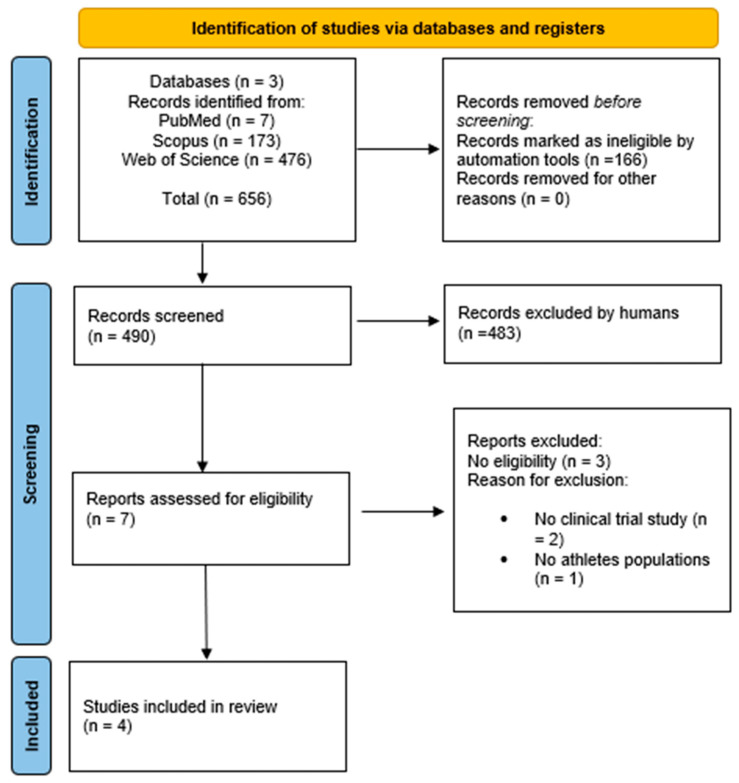
The PRISMA flow diagram of the study selection process. The final actual number of included studies is four.

A total of 656 articles were identified, and after excluding duplicates (166) using the Rayyan system, 490 articles remained. Among these, 483 were excluded following title and abstract screening. The remaining seven articles were screened in full text. Of these seven articles, three did not have data on the effects of sport activity on sperm parameters and were therefore excluded. The flowchart presented in Figure 1 provides a detailed description of the research selection process.

The four included studies comprised a total of 156 participants with ages ranging from 15 to 30 years old. All included studies evaluated traditional parameters (volume, motility, morphology, and concentration). Moreover, each included study evaluated different components of semen that could affect fertility, like sperm DNA fragmentation [56], seminal ROS [49], and seminal cytokines [48]. One study assessed the hormonal aspect [56], and one study assessed testicular volume in athletes with varicocele [57].

The study developed by Radojevic et al. [57] focused on testing the hypothesis that stopping sports training in young athletes reduces the prevalence of varicocele and related seminal parameters, leading to male infertility [57]. The second article focused on the analysis of semen parameters, especially sperm DNA fragmentation and hormone parameters, for high-level triathletes [56]. The third article focused on the effects of 16 weeks of intensive cycling training on semen parameters, especially seminal ROS, malondialdehyde, superoxide dismutase, catalase, and total antioxidant capacity in male road cyclists [49]. And the last article focused on the effects of long-term low-to-intensive cycling training on semen parameters and seminal cytokines [48].

The results of the studies included within this systematic review are summarized in Table 3, where different study characteristics are also reported: first author surname, year of publication, number of participants, age, sporting discipline of appurtenance, type of physical activity, purpose, outcomes (with a subsection reporting the seminal parameters measured), and principal results.

The study of Radojevic et al. [57] evaluated the semen parameters after stopping sports practice in young non-professional athletes affected by varicocele, divided into two groups, namely, 52 BFVH (basketball, football, volleyball, and handball) players and 4 water polo players and a control group of 40 inactivity individuals. Seminal parameters were measured at baseline and after a 6-month stop from sporting activity. The results showed that in the BFVH group, after a 6-month interruption from sporting activity, seminal fluid analysis showed improvements in all parameters measured with statistical significance for sperm concentration (*p* < 0.001) and progressive motility (*p* < 0.023). No significant differences in seminal fluid parameters were found in the water polo group after the 6-month break. The percentage of young males displaying any of the four grades of varicocele (including subclinical cases), at 17.05%, was highest in the BFVH group. This rate was significantly higher (Zi = 1.986, *p* < 0.049) compared to the control group, which had a rate of 12.35%. Although basketball players exhibited the highest percentage of varicoceles at 18.68%, no significant differences were found between the various sports. When comparing the varicocele grades between individuals in the BFVH group and those in the non-sport active group, no significant difference was observed in the distribution of grades. However, the nominal difference was most notable when comparing the third grade: 11.53% in the BFVH group versus 7.31% in the non-sport active young males; Zi = 0.83, *p* > 0.05. The testicular volume in BFVH was lower than the reference value but remained within the normal range in water polo players as they had a subclinical or first degree of varicocele.

In the second article included, Vaamonde et al. [56] analyzed semen parameters, in particular, DNA fragmentation and hormonal parameters in 12 elite triathletes. Semen samples were obtained during the preparation period for the long-distance National Triathlon Championship. Prior to the competition, the triathletes underwent a typical 2-week period of lowered training volume known as “tapering” and were compared with their normal range [58]. The semen analysis results indicated that the parameters were within normal ranges, including sperm concentration, motility, and morphology. However, DNA fragmentation was found to be elevated, with an average value of 20.4 ± 6.1%. Additionally, the presence of round cells in the semen was higher than normal, and some athletes exhibited elevated numbers of active macrophages. While sperm morphology remained within the normal range, it was close to the lower reference value (5.3 ± 2.7), with some athletes showing values below the normal threshold. Hormonal levels were within normal ranges for all individuals examined. The study revealed significant correlations between several parameters. Specifically, the presence of round cells showed a significant correlation with progressive motility, sperm morphology, and DNA fragmentation. Furthermore, DNA fragmentation was also correlated with the testosterone/cortisol (T/C ratio). No other significant associations were found [56].

In the third article included, Maleki et al. [49] examined the effects of 16 weeks of intensive training on seminal parameters, including seminal ROS, malondialdehyde (MDA), superoxide dismutase (SOD), catalase, and total antioxidant capacity (TAC), in 24 male road cyclists. The semen samples were collected, respectively, at baseline (T1), immediately (T2), 12 (T3), and 24 (T4) hours after the last training session in week 8; immediately (T5), 12 (T6), and 24 (T7) hours after the last training session in week 16; and 7 (T8) and 30 (T9) days after the last training session in week 16. Concerning the sperm morphology, significant differences were observed at T3–T7 than baseline (*p* < 0.008). Semen volume, sperm concentrations, and sperm motility improved to their baseline values after 7 days of recovery. However, sperm morphology and number of spermatozoa remained significantly low after 30 days of recovery (*p* < 0.008). The decreases in semen volume, sperm concentrations, sperm motility, and number of spermatozoa were significant at T2–T7 than baseline (*p* < 0.008). The results showed that levels of seminal ROS and MDA increased after 16 week of training (*p* < 0.008) and remained high after 30 days of recovery. The levels of seminal SOD, catalase, and TAC decreased (*p* < 0.008) and remained low after 30 days of recovery (*p* < 0.008). The decreases in semen volume, sperm concentrations, sperm motility, and number of spermatozoa were significant at T2–T7 than baseline (*p* < 0.008) [49].

In the last study included, Maleki et al. [48] evaluated the effects of long-term low-to-intensive cycling training on semen parameters, especially seminal cytokines in 24 male road cyclists. The semen samples were collected, respectively, at baseline (T1), immediately (T2), 12 (T3), and 24 (T4) hours after the last training session in week 8; immediately (T5), 12 (T6), and 24 (T7) hours after the last training session in week 16; as well as 7 (T8) and 30 (T9) days after the last training session in week 16. Regarding variations in seminal parameters semen volume, sperm concentrations, sperm motility, number of spermatozoa, and sperm morphology were significant decreased at T2–T7 than baseline (*p* < 0.008). Semen volume, sperm concentrations, and sperm motility improved to their baseline values after 7 days of recovery. However, sperm morphology and number of spermatozoa remained significantly low after 30 days of recovery (*p* < 0.008). The authors showed that seminal plasma IL-1b, IL-6, IL-8, and TNF-a increased significantly at T2–T7 when compared with baseline values (*p* < 0.008). IL-1b, IL-6, and IL-8 values remained significantly high after 30 days of recovery (*p* < 0.008). TNF-a values returned to the baseline values after 30 days of recovery [48].

To summarize the findings of the studies included in our review, the results showed that professional athletes, particularly elite triathletes, had semen parameters within the physiological range. However, morphological values were close to the lower reference limit, with some athletes having values below the minimum reference limit. In particular, these professional athletes had high levels of sperm DNA fragmentation. In contrast, non-professional athletes showed alterations in traditional semen parameters, as well as elevated levels of reactive oxygen species (ROS), malondialdehyde (MDA), and pro-inflammatory interleukins, and reduced levels of superoxide dismutase (SOD), catalase, and total antioxidant capacity (TAC). In non-professional athletes with varicocele, semen parameters and testicular volume were found to have improved compared to baseline in the BFVH group, but not in the W group, after six months of cessation of athletic activity. Our results showed that in both professional and non-professional athletes, with or without varicocele, semen parameters improved after a period of rest from sport.

## 4. Discussion

In this systematic review, we performed a detailed analysis of the current literature on the semen parameters in professional and non-professional athletes. Only studies on athletes with an intensive training regime were included. The studies present a remarkable heterogeneity: one study [56] was observational while the other three were intervention studies [48,49,57]. Only two studies performed tests to assess the physical condition of the participants [48,49]. Moreover, in three studies [48,49,56], the seminal fluid was evaluated according to WHO guidelines [58], while in the other [57], they were evaluated according to the criteria given by the Guidelines on Male Infertility 2010 [59]. Seminal fluid was correctly collected following a period of sexual abstinence ranging from 2 to 5 days in all studies. The seminal parameters consistently measured across these studies included semen volume, sperm motility, sperm morphology, and sperm concentration. It should be noted that in 2021, the WHO published a new laboratory manual for the examination and processing of human semen [13]. Therefore, the studies included in this review used the previous edition of the manual published in 2010 [58].

Radojevic et al. [57] described testicular volume; Maleki et al. [49] evaluated seminal plasma oxidative stress biomarkers (ROS and MDA) and antioxidants (SOD, catalase, and TAC); Maleki et al. [48] evaluated seminal plasma cytokines (IL-1b, IL-6, IL-8, and TNF-a); and the last included study [56] evaluated DNA sperm damage. Only one study conducted a hormone assessment of athletes. Our results showed that sporting activity could affect the quality of seminal fluid differently. In the scientific literature, we have few studies evaluating the relationship between sport and seminal fluid; most studies focus on the correlation between physical activity and seminal parameters rather than on sporting activity. This systematic review only includes studies on professional and non-professional athletes, with the aim of assessing the extent to which the high training regime could influence seminal parameters and thus male fertility.

Physical activity is a cornerstone of health and well-being, according to the WHO. Regular physical activity improves a person’s overall health by reducing the risk of major non-communicable diseases like heart disease, stroke, diabetes, and cancer, while promoting a healthy weight, stronger bones and improved physical function [60]. The WHO defines physical activity as any bodily movement produced by skeletal muscles that requires energy expenditure. Physical activity refers to all movement, including movement during leisure time, for transport to get to and from places, or as part of a person’s work [60]. In contrast, physical exercise is defined as a set of bodily movements performed in a planned and repeated manner to maintain or improve physical fitness [60]. Jóźków et al. [52] showed that high levels of physical activity can increase the percentage of immobile spermatozoa; in contrast, Matorra et al. [61] showed that high levels of physical activity improve in vitro fertilization rates. All in all, these two sperm donor studies [52,61] used the IPAQ to estimate physical activity and thus the levels of physical activity were self-reported. So, we do not really know how much exercise impacts male fertility. Maleki et al. [39] showed that moderate-intensity exercise (MICT), high-intensity exercise (HICT), and high-intensity interval exercise (HIIT) over a 24-week period led to a reduction in markers of oxidative stress and inflammation in seminal fluid, with different response times for each type of exercise. These changes were associated with improvements in seminal fluid quality parameters and sperm DNA integrity in healthy individuals. MICT was found to be more effective than HICT and HIIT in improving markers of male reproductive function, indicating that a regular training program of moderate intensity may lead to beneficial adaptations in male fertility [49]. Different studies [48,49] showed that 16 weeks of intense cycling had a negative effect on the seminal parameters of the participants, in particular, a significant decrease in seminal volume, sperm concentration, sperm motility, and sperm morphology, but after a week of rest, the parameters returned almost to their initial levels except for sperm morphology and sperm concentration, which were still low after 30 days of rest. Wise et al. [50] showed that cycling for more than five hours a week was associated with low sperm concentration. While another study [62] showed that endurance cycling training performed at least three times a week for 40 min for session did not lead to significant differences in sperm motility, volume, and sperm concentration; however, cyclists were found to have lower percentages of sperm with normal morphology than the control group.

**Figure 2:**
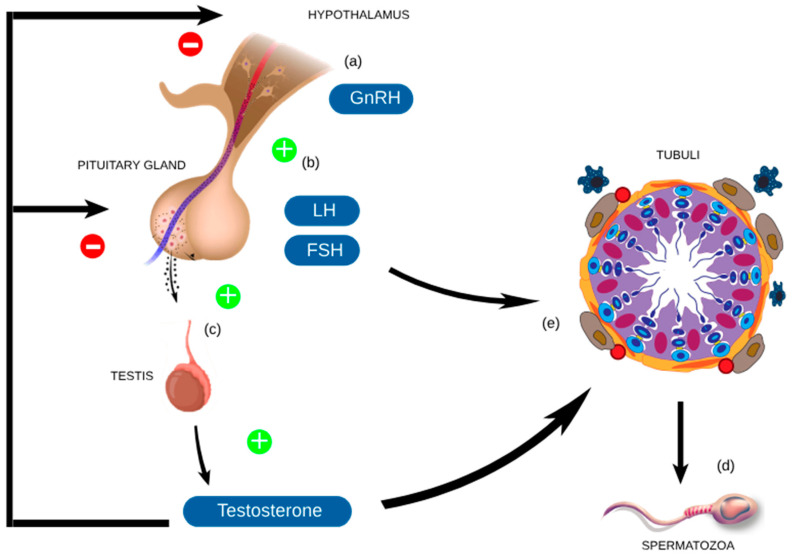
The figure illustrates the spermatogenesis process. Gonadotropin-releasing hormone (GnRH) is released by hypothalamus (**a**), which stimulates the anterior pituitary gland (**b**) to secrete LH and follicle-stimulating hormone (FSH). LH stimulates the testes (**c**) to produce testosterone, whereas FSH supports the functionality of the seminiferous tubules (**e**) along with testosterone. The process proceeds through several stages until mature spermatozoa (**d**) are released from tubules (**e**). The process is regulated through a feedback mechanism.

Maleki showed that 16 weeks of intensive cycling training increased levels of sperm oxidants (ROS, MDA) and decreased levels of anti-oxidants (SOD, TAC, catalase) [49]. Extensive evidence highlights the significant role of oxidative stress in male infertility development [18,19,20]. Oxidative stress can damage the spermatozoa membrane and DNA, decreasing their fertilization capability and the potential to produce a healthy embryo [23,28,29,30,31]. These findings emphasize the importance of maintaining a balance between ROS and antioxidants to ensure male reproductive health. Moreover, the compression on the scrotum, produced mainly by the bike saddle and the tight clothes worn, may generate an undesired increase in intra scrotal temperature, which is well known to be detrimental to sperm production [63]. Any increase in scrotal temperature can disrupt spermatogenesis by causing germ cell death through mechanisms like autophagy, DNA damage, and apoptosis [64]. Everyday clothing is thought to influence spermatogenesis: tight trousers generate more heat for the testes than loose trousers, with walking seemingly reducing this effect due to increased peri genital air circulation [65]. The question of whether more physical activity leads to higher scrotal temperature is still under discussion [66,67]. Maleki et al. [48] showed that 16-week low-to-intensive cycling training may have deleterious consequences for spermatozoa and hence may have an impact on male fertility among cyclists, as levels of seminal IL-1b, IL-6, and IL-8 increased and remained high after 30 days of recovery. In contrast, Fitzgerald et al. [68] showed that plasma IL-6 levels were four times lower in amateur triathletes and two times lower in amateur cyclists than in recreational athletes and lower in triathletes than in cyclists. Regarding triathlon, one of the studies included in this systematic review [56] showed that in triathletes DNA fragmentation showed high values. Different studies suggest that sperm DNA fragmentation is associated with reduced male fertility [69,70]. Furthermore, the presence of round cells in the sperm was higher than normal, and some of the athletes showed a high number of activated macrophages. The spermatogenesis process is reported in Figure 2. Although the morphology was within normal values, close to the lowest reference value, and some of the athletes had values below the normal level [56].

In triathlon athletes, Vaamonde et al. [71] showed a high inverse correlation between the percentage of spermatozoa with normal morphology and weekly cycling volume, concluding that high cycling volume, especially if it exceeds 300 km/week, is detrimental to sperm morphology and can lead to severe impairment of male fertility.

The study of Vaamonde et.al [56] is the only study included analyzing hormonal parameters in athletes in this review: a positive correlation between DNA fragmentation and T/C ratio [56]. Based on these findings, future research evaluating hormonal parameters are needed.

Radojevic et al. [57] showed how 6-mounths stop of sporting activity went to improve seminal parameters in varicocele-affected athletes. After 6 months of stop athletes of various discipline affected by varicoceles significantly improved sperm concentration and progressive motility, while sperm morphology, volume, and testicular volume did not change. The results showed that the percentage of young males exhibiting any of the four grades of varicocele (including the subclinical form) was highest in the BFVH group (basketball, football, volleyball, and handball); in addition, basketball players showed the highest percentage of varicocele, along with the prevalence of higher grades (second and third) between the different sports, while the water polo players showed the lowest incidence. The authors hypothesize that typical jumping against flat gravitational forces, as found in basketball, increases the presence of varicocele, given the direction of venous blood from the testes. The findings clearly indicate that stopping athletic activity can improve sperm parameters in young males with varicocele. Therefore, indirectly, the sports undertaken by the young males from the BFVH group did contribute to their increased rates of varicocele. Indeed, the participants had been involved in sports for an average of three and a half years before the study. Therefore, the question remains as to what extent seminal fluid quality can be reversed in athletes who have been engaged in sports for more than five years.

Sports-related varicocele in pubescent males is a condition that can have a positive prognosis when diagnosed early and managed with a reduction in sports activity. Semen parameters critical to fertility improve after a break from sports training. Moreover, the results of this study contrast with those of Rigano et al. [72], who concluded that sport training does not alter the prevalence of varicocele compared to the general population, although physical activity should still be considered an aggravating factor in the natural progression of varicocele. Considering the overall benefits of physical activity, young males with varicocele should engage in sports that do not increase the risk of the condition. According to Zampieri et al. [73] et al. in athletes with subclinical varicocele, sports activity could worsen progression to clinical varicocele. In support of the hypothesis, Di Luigi et al. [74] showed that athletes with varicocele had a significantly reduced percentage of normal spermatozoa and a significantly reduced progressive forward motility than the group of non-athletes with varicocele. However, the included studies after being reviewed presented different biases. First, one of the most important problems is that the number of participants in the different manuscripts is quite different. Then, another issue is the absence of control groups. The only included study that used a control group was Radojevic’s study [57] of athletes with varicocele. However, the groups were not homogeneous [57]. Another issue is the hormonal assessment, present only in the study of Vaamonde et al. [56]. The main problem is that in the scientific literature, we have little and poorly structured data concerning this issue. Ideally, the seminal parameters, hormonal parameters, and other different parameters related to male fertility in athletes at different stages of sports programming have to be evaluated. In addition, future studies should include more measurements at different time periods to evaluate how effectively sport activity affects male fertility to compare fertility-related parameters with the minimum values reported by the guidelines, and one could also compare variation over time.

## 5. Conclusions

Intensive training may worsen seminal parameters and, consequently, male fertility. Due to the limited number of papers selected and the considerable variation in protocols, the certainty of evidence is very low, and the results should be interpreted with caution.

## Figures and Tables

**Table 1:** Lower reference limits (5th centiles and their 95% confidence intervals) for semen parameters.

Parameter	Unit of Measurement	Reference Value (5th Centiles)	(95% CI)
Semen volume	mL	1.4	(1.3–1.5)
Sperm concentration	106 per mL	16	(15–18)
Total sperm number	106 per ejaculate	39	(35–40)
Total motility	%	42	(40–43)
Progressive motility	%	30	(29–31)
Non-progressive motility	%	1	(1–1)
Immotile spermatozoa	%	20	(19–20)
Vitality	%	54	(50–56)
Normal forms	%	4	(3.9–4.0)

**Table 2:** Search strategy carried out in each database.

Database	Query	Results
PubMed	((“semen quality” [All Fields] OR “sperm” [All Fields] OR “seminal quality” [All Fields] OR “semen motility” [All Fields] OR “semen volume” [All Fields] OR “sperm parameters” [All Fields] OR “spermatozoa” [All Fields] OR “sperm concentration” [All Fields] OR “male reproduction” [All Fields]) AND (“trained” [All Fields] OR “athletes elite” [All Fields] OR “amateur” [All Fields] OR “athletes” [All Fields] OR “sport” [All Fields]) AND ((“clinical trial” [Publication Type] OR “randomized controlled trial” [Publication Type]) AND 2014/01/01:2024/12/31 [Date—Publication]) AND “clinical trial” [Publication Type]) AND (clinical trial [Filter])	7
Scopus	(TITLE-ABS-KEY (semen AND quality) OR TITLE-ABS-KEY (sperm) OR TITLE-ABS-KEY (seminal AND quality) OR TITLE-ABS-KEY (semen AND motility) OR TITLE-ABS-KEY (semen AND volume) OR TITLE-ABS-KEY (sperm AND parameters) OR TITLE-ABS-KEY (spermatozoa) OR TITLE-ABS-KEY (sperm AND concentration) OR TITLE-ABS-KEY (male AND reproduction) AND TITLE-ABS-KEY (athletes AND elite) OR TITLE-ABS-KEY (amateur) OR TITLE-ABS-KEY (sport) OR TITLE-ABS-KEY (athletes) OR TITLE-ABS-KEY (trained)) AND PUBYEAR > 2013 AND PUBYEAR < 2025 AND (LIMIT-TO (DOCTYPE, “ar”)) AND (LIMIT-TO (SUBJAREA,”MEDI”)) AND (LIMIT-TO (LANGUAGE, “English”)) AND (LIMIT-TO (EXACTKEYWORD, “Article”))	173
Web of Science (Medline)	(((((((((TS=(semen quality)) OR TS=(sperm)) OR TS=(seminal quality)) OR TS=(semen motility)) OR TS=(semen volume)) OR TS=(sperm parameters)) OR TS=(spermatozoa)) OR TS=(sperm concentration)) OR TS=(male reproduction)) AND (((((TS=(athletes elite)) OR TS=(sport)) OR TS=(amateur)) OR TS=(athletes)) OR TS=(trained)) and 2014 or 2015 or 2016 or 2017 or 2018 or 2019 or 2020 or 2021 or 2022 or 2023 or 2024 (Publication Years) and Journal Article (Publication Type) and Male (MeSH Headings) and English (Languages)	476

**Table 3:** Results of included studies.

Author	Participants (N)	Age (Years)	Type of Sport	Type and/or Frequency of Exercise	Purpose	Fertility Outcomes	Results
Radojevic et al., 2015 [57]	Group A: 52 Group B: 4 Group C: 40	Group A: 16.5 ± 0.55 Group B: 15.9 ± 0.78 Group C: 16.1 ± 0.24	Group A: football, basketball, volleyball and handball Group B: Water polo Group C: Inactivity	Group A and B: At least three training sessions a week in 3 years Group C: Occasional training, i.e., one or less training session per week	To evaluate the cessation of sports training in young athletes reduces the prevalence of varicocele and related infertility parameters	Sperm parameters (sperm concentration, progressive motility, sperm morphology, ejaculate volume), varicocele test (ultrasonography), and volume of left testicle	Group A showed an improvement in all parameters of seminal fluid analysis. Group B showed no significant differences in the sperm parameters tested were found.
Vaamonde et al., 2018 [56]	12 High-level triathletes	27 ± 3	Triathlon	Preparation period for the long-distance National Triathlon Championship/Tapering training protocol	To analyze semen and hormone parameters in elite triathletes	Sperm parameters (volume, concentration, total motility, total sperm number, non-progressive motility, sperm morphology, DNA fragmentation, round cells) and hormone parameters (testosterone, cortisol, and T/C ratio)	Semen volume, concentration and motility were within normal ranges. Morphology was within normal values, i.e., it was close to the lower reference value, and some of the athletes had values below the normality level. DNA fragmentation showed high values. Hormonal values were within normal ranges.
Maleki et al., 2014 [49]	24 Non-professional cyclists	23.1 ± 6.2	Cycling	16 weeks of intensive cycling training	To examine the effects of 16 weeks of intensive cycling training on seminal parameters, especially reactive oxygen species (ROS), malondialdehyde (MDA), superoxide dismutase (SOD), and total antioxidant capacity (TAC)	Sperm parameters (semen volume, motility, sperm morphology, sperm concentration sperm concentration, ROS, MDA, SOD, TAC, and catalase)	Significant changes were noted in traditional semen parameters. After a 7-day recovery period, semen volume, concentrations, and motility returned to baseline values. Sperm morphology and concentration remained significantly lower even after 30 days of recovery. The levels of seminal ROS and MDA increased and remained high after 30 days of recovery. The levels of seminal SOD, catalase, and TAC decreased and remained low after 30 days of recovery.
Maleki et al., 2015 [48]	24 Non-professional cyclists	23.1 ± 6.2	Cycling	16 weeks of long-term low-to-intensive cycling training	To examine the effects of long-term low-to-intensive cycling training on semen parameters and seminal cytokines	Sperm parameters (semen volume, sperm motility, sperm morphology, sperm concentration, number of spermatozoa, seminal interleukin (IL)-1β, IL-6, IL-8, and tumor necrosis factor-α)	Traditional sperm parameters decreased. All of the above-mentioned variables (with the exception of semen volume, sperm motility, and sperm concentration) remained low after 30 days of recovery. The seminal interleukin levels increased and remained high after 30 days of recovery.

## Supplementary Materials

The following supporting information can be downloaded at https://www.mdpi.com/article/10.3390/jcm14010201/s1.
