# Supplementary material for: The Influence of an Intense Training Regime in Professional and Non-Professional Athletes on Semen Parameters: A Systematic Review
Source: J Clin Med. 2025 Jan 1;14(1):201. doi: 10.3390/jcm14010201 (PMC11720830; doi:10.3390/jcm14010201)
Supplement: Supplementary file 1 [file jcm-14-00201-s001.zip › jcm-3372318-supplementary.pdf]

Nemanja Radojevic 2015

N.	CRITERIA	YES	NO	Other (CD, NR, NA)*
1	1. Was the study described as randomized, a randomized trial, a randomized clinical trial, or an RCT?			**NR**
2	2. Was the method of randomization adequate (i.e., use of randomly generated assignment)?	**X**		
3	3. Was the treatment allocation concealed (so that assignments could not be predicted)?		**X**	
4	4. Were study participants and providers blinded to treatment group assignment?		**X**	
5	5. Were the people assessing the outcomes blinded to the participants' group assignments?		**X**	
6	6. Were the groups similar at baseline on important characteristics that could affect outcomes (e.g., demographics, risk factors, co-morbid conditions)?	**X**		
7	7. Was the overall drop-out rate from the study at endpoint 20% or lower of the number allocated to treatment?			**NR**
8	8. Was the differential drop-out rate (between treatment groups) at endpoint 15 percentage points or lower?			**NR**
9	9. Was there high adherence to the intervention protocols for each treatment group?	**X**		
10	10. Were other interventions avoided or similar in the groups (e.g., similar background treatments)?	**x**		
11	11. Were outcomes assessed using valid and reliable measures, implemented consistently across all study participants?	**x**		
12	Did the authors report that the sample size was sufficiently large to be able to detect a difference in the main outcome between groups with at least 80% power?			**NR**
13	13. Were outcomes reported or subgroups analyzed prespecified (i.e., identified before analyses were conducted)?	**x**		
14	4. Were all randomized participants analyzed in the group to which they were originally assigned, i.e., did they use an intention-to-treat analysis?	**x**		

Radojevic N, Radunovic M, Pajovic B. Restricting sports activity in reducing the rate of varicocele and related infertility parameters in athletes. Arch Med Sci. 2015 Mar 16;11(1):169-73. doi: 10.5114/aoms.2015.48146. Epub 2015 Jan 8. PMID: 25861305; PMCID: PMC4379365.

Maleki 2014

N.	CRITERIA	YES	NO	Other (CD, NR, NA)*
1	1. Was the study described as randomized, a randomized trial, a randomized clinical trial, or an RCT?			**NA**
2	2. Was the method of randomization adequate (i.e., use of randomly generated assignment)?			**NA**
3	3. Was the treatment allocation concealed (so that assignments could not be predicted)?			**NA**
4	4. Were study participants and providers blinded to treatment group assignment?		**X**	
5	5. Were the people assessing the outcomes blinded to the participants' group assignments?		**X**	
6	6. Were the groups similar at baseline on important characteristics that could affect outcomes (e.g., demographics, risk factors, co-morbid conditions)?	**x**		
7	7. Was the overall drop-out rate from the study at endpoint 20% or lower of the number allocated to treatment?			**NR**
8	8. Was the differential drop-out rate (between treatment groups) at endpoint 15 percentage points or lower?			**NA**
9	9. Was there high adherence to the intervention protocols for each treatment group?	**X**		
10	10. Were other interventions avoided or similar in the groups (e.g., similar background treatments)?	**x**		
11	11. Were outcomes assessed using valid and reliable measures, implemented consistently across all study participants?	**x**		
12	Did the authors report that the sample size was sufficiently large to be able to detect a difference in the main outcome between groups with at least 80% power?			**NR**
13	13. Were outcomes reported or subgroups analyzed prespecified (i.e., identified before analyses were conducted)?		**x**	
14	14. Were all randomized participants analyzed in the group to which they were originally assigned, i.e., did they use an intention-to-treat analysis?			**NA**

Maleki BH, Tartibian B, Vaamonde D. The effects of 16 weeks of intensive cycling training on seminal oxidants and antioxidants in male road cyclists. Clin J Sport Med. 2014 Jul;24(4):302-7. doi: 10.1097/JSM.0000000000000051. PMID: 24389625.

Maleki 2015

N.	CRITERIA	YES	NO	Other (CD, NR, NA)*
1	1. Was the study described as randomized, a randomized trial, a randomized clinical trial, or an RCT?		**x**	
2	2. Was the method of randomization adequate (i.e., use of randomly generated assignment)?			**NA**
3	3. Was the treatment allocation concealed (so that assignments could not be predicted)?			**NA**
4	4. Were study participants and providers blinded to treatment group assignment?		**X**	
5	5. Were the people assessing the outcomes blinded to the participants' group assignments?		**X**	
6	6. Were the groups similar at baseline on important characteristics that could affect outcomes (e.g., demographics, risk factors, co-morbid conditions)?	**x**		
7	7. Was the overall drop-out rate from the study at endpoint 20% or lower of the number allocated to treatment?			**NR**
8	8. Was the differential drop-out rate (between treatment groups) at endpoint 15 percentage points or lower?			**NA**
9	9. Was there high adherence to the intervention protocols for each treatment group?	**X**		
10	10. Were other interventions avoided or similar in the groups (e.g., similar background treatments)?	**x**		
11	11. Were outcomes assessed using valid and reliable measures, implemented consistently across all study participants?	**x**		
12	Did the authors report that the sample size was sufficiently large to be able to detect a difference in the main outcome between groups with at least 80% power?			**NR**
13	13. Were outcomes reported or subgroups analyzed prespecified (i.e., identified before analyses were conducted)?		**x**	
14	14. Were all randomized participants analyzed in the group to which they were originally assigned, i.e., did they use an intention-to-treat analysis?			**NA**

Hajizadeh Maleki B, Tartibian B. Long-term Low-to-Intensive Cycling Training: Impact on Semen Parameters and Seminal Cytokines. Clin J Sport Med. 2015 Nov;25(6):535-40. doi: 10.1097/JSM.0000000000000122. PMID: 24977955.

N.	CRITERIA	YES	NO	Other (CD, NR, NA)*
1	1. Was the study described as randomized, a randomized trial, a randomized clinical trial, or an RCT?			**NR**
2	2. Was the method of randomization adequate (i.e., use of randomly generated assignment)?			**NA**
3	3. Was the treatment allocation concealed (so that assignments could not be predicted)?			**NA**
4	4. Were study participants and providers blinded to treatment group assignment?		**X**	
5	5. Were the people assessing the outcomes blinded to the participants' group assignments?		**X**	
6	6. Were the groups similar at baseline on important characteristics that could affect outcomes (e.g., demographics, risk factors, co-morbid conditions)?	**X**		
7	7. Was the overall drop-out rate from the study at endpoint 20% or lower of the number allocated to treatment?			**NR**
8	8. Was the differential drop-out rate (between treatment groups) at endpoint 15 percentage points or lower?			**NA**
9	9. Was there high adherence to the intervention protocols for each treatment group?	**X**		
10	10. Were other interventions avoided or similar in the groups (e.g., similar background treatments)?	**x**		
11	11. Were outcomes assessed using valid and reliable measures, implemented consistently across all study participants?	**x**		
12	Did the authors report that the sample size was sufficiently large to be able to detect a difference in the main outcome between groups with at least 80% power?			**NR**
13	13. Were outcomes reported or subgroups analyzed prespecified (i.e., identified before analyses were conducted)?	**x**		
14	14. Were all randomized participants analyzed in the group to which they were originally assigned, i.e., did they use an intention-to-treat analysis?			**NA**

Vaamonde D, Algar-Santacruz C, Abbasi A, García-Manso JM. Sperm DNA fragmentation as a result of ultra-endurance exercise training in male athletes. Andrologia. 2018 Feb;50(1). doi: 10.1111/and.12793. Epub 2017 Mar 15. PMID: 28295487.
